# Comparison of Quality of Life and Work Ability of Taxi and Motorcycle Taxi Drivers: Evidence from Brazil

**DOI:** 10.3390/ijerph16040666

**Published:** 2019-02-25

**Authors:** Hugo Machado Sanchez, Eliane Gouveia de Morais Sanchez, Maria Alves Barbosa, Celmo Celeno Porto, Mario Silva Approbato

**Affiliations:** 1 Universidade de Rio Verde (UniRV), Fazenda Fontes do Saber, 75901-970 Rio Verde, Goiás, Brazil; 2 Universidade Federal de Goiás (UFG), Rod BR 364 KM 192—Setor Parque Industrial No. 3800, 75801-615 Jataí, Goiás, Brazil; egmfisio@yahoo.com.br; 3 Universidade Federal de Goiás (UFG), R. 235, s/n-Setor Leste Universitário, 74605-050 Goiânia, GO, Brazil; maria.malves@gmail.com (M.A.B.); celmo1934@gmail.com (C.C.P.); approbato.m@hotmail.com (M.S.A.)

**Keywords:** quality of life, work, occupation, transportation, health

## Abstract

Urban transport drivers, specifically taxi and motorcycle taxi drivers, are exposed to particular environmental, societal, and health situations related to their occupation. To compare work capacity and quality of life of taxi and motorcycle taxi drivers, and correlate quality of life and work ability, a cross-sectional descriptive study was conducted among 232 motorcycle taxi drivers and 60 male taxi drivers in urban cities of Brazil. Three instruments were used for evaluation: a questionnaire on sociodemographic and occupational issues, the Work Capacity Index (WCI), and the WHOQOL-bref (World Health Organization Quality of Life–Bref). Taxi drivers presented better evaluation scores in the physical and psychological domains and general quality of life (QOL) (*p* < 0.01), and better self-perceived work ability (lower physical and mental demands, fewer diagnosed diseases, less incapacity for professional practice, *p* < 0.001). In addition, there was a positive relationship between QOL and WCI (*p* = 0.001). Motorcycle taxi drivers had worse self-perceived QOL and of work ability, and there was a positive correlation between QOL and work ability.

## 1. Introduction

Urban transport systems are essential to the functioning of modern cities. They facilitate transportation of the population by providing an alternative means of carrying out their daily tasks, thus influencing social and economic dynamics. However, urban transport workers, among them taxi and motorcycle taxi drivers, are vulnerable to the precarious work conditions offered by the environment and, consequently, are susceptible to the development of health problems associated with their occupation. Studies related to worker health frequently address two terms: work ability (WA) and quality of life (QOL) directly related to subjective well-being [1].

The concept of WA is multidimensional; it involves self-perceptions of physical, mental, and social conditions as a context for questions on issues such as the health, work, and lifestyle of the individual. According to specialists in the area, WA is the balance between work demands and unstable personal resources whose purpose is to improve professional performance over a longer period [2]. QOL can be defined as an individual’s conception of their position in life so that the individual relates his or her culture and values to his or her goals, expectations, standards, and concerns [1]. The WA and QOL of taxi and motorcycle taxi drivers are factors in determining their working conditions, which are influential in their predisposition to develop chronic diseases [1,2].

In the face of contemporary needs, the global trend is towards seeking alternative transport methods. This has led to an increase in the number of professionals related to this sector, more specifically taxi and motorcycle taxi drivers. However, these are working populations that do not receive due attention from health and labor agencies, leaving a gap in health and occupational policies [3,4]. This fact is also due to the low interest in research about this population, with investigations mainly examining quantitative accident data, leaving other important variables, such as QOL and WA, without due scientific attention. The evaluation of QOL includes health (musculoskeletal pain, poor diet, hypertension, diabetes, fatigue, physical inactivity, obesity, and constipation), psychological (anxiety, stress, and mental capacity), environmental (work instruments, physical security, protection, financial resources, health care/social, opportunities to acquire new information/skills, participation in recreation/leisure, and physical environment), and social (psychosocial well-being, individual capacity, working hours, absence of rest, and risk of accident) issues, while the analysis of WA involves the demands of the profession (physical and mental), the projected state of health of the workers, and the capacity to carry out their work activities in the present and in the future [1,2,3,4,5,6,7,8,9,10,11,12]. In the context of increasingly long workdays with less physical fitness, the study of WA has become an indicator allowing evaluation of WA from the perception of the worker him or herself through pre-established questions in contexts in which the worker is included in his or her work activity [10].

Taxi and motorcycle taxi drivers in Brazil have poor self-perceived health, with significant numbers of work accidents and absences, job insecurity, and lack of social security. Taxi driving is considered a sub-profession. Investments in health education programs must be directed at these professionals to make them aware of the occupational hazards of their profession and thereby promote their interest in measures aimed at reducing the need for future expenses related to primary care treatment or rehabilitation [5,6,7,8,9,10].

Most taxi and motorcycle taxi drivers are young male adults who, because of their work, are exposed to many factors that impact their health and, consequently, their QOL and WA. Inadequate variation in posture during work, mainly because of the impossibility of regular and predictable breaks, causes taxi and motorcycle taxi drivers to experience back injuries and pain, which lead to many cases of physical disability [4].

The QOL of taxi drivers in Brazil can be influenced by factors related to their well-being, and this profession often functions on an informal basis. Some drivers work in situations where they are constantly exposing themselves to adverse conditions in order to increase productivity and profitability. These conditions may result in multiple injuries that can generate occupational stress and negatively impact on health/disease processes [5,6].

Motorcycle taxi drivers were considered an alternative transportation service for many years, but were subsequently recognized as a professional and profitable activity and regulated as a professional passenger transport activity. Motorcycle taxis are common, especially in developing or underdeveloped countries such as Brazil, Mexico, Thailand, and other nations in South America, Africa, and Asia [3,4,5,6,7,8,9,10,11,12].

There is a global trend to seek alternative means of public transportation; therefore, an increase in the number of professionals in this sector—more specifically, taxi and motorcycle taxi drivers—is expected. However, they belong to working populations that do not receive attention from health and labor offices and are excluded from health and occupational policies [3,4,5,6,7,8]. The latter may also be attributed to the low interest researchers have in these professionals, conducting mostly quantitative investigations of accidents and not studying other important variables, such as QOL and work capacity. The evaluation of QOL involves a broad spectrum of health, psychological, environmental, and social issues, and the analysis of work capacity shows professional demands (physical and mental), health status of workers, and their ability to perform work activities in the present and future [4,5,6,7].

Studies on the abovementioned areas are scarce and there are no data on which of these two working populations has higher needs related to items analyzed through QOL and WA. Organizations that regulate and protect health and workers’ rights possess limited information to be able to propose public health policies and provide specific assistance to these working populations [5,6,7,8,9,10]. 

A study among taxi drivers in the state of Bahia, Brazil, concluded that the occurrence of traffic accidents is significantly related to the presence of lower-limb fatigue and musculoskeletal complaints [6,7,8,9]. These complaints were due to the precarious work conditions of taxi drivers. For example, they did not have an adequate place to rest/or take a break in taxi stands or a space where they could recover from physical and postural capacities affected by working conditions, contributing to postural and muscular injuries that could impact their general health [9]. Sedentarism is the main risk factor that triggers chronic non-communicable diseases in professions with long working hours [10]. A range of occupational risks prevents taxi and motorcycle taxi drivers from living a healthy life [4]. The sometimes long and uninterrupted workdays make it impossible for drivers to have regular intervals or rest breaks; thus, they become susceptible to diseases. Workdays without regular intervals also expose workers to negative eating conditions [5,6,9,10]. In addition, the exhaustive average daily workload of up to 11 hours is closely related to the decrease in the QOL and WA of these workers [5].

As mentioned, taxi and motorcycle taxi drivers’ occupations have both common and unique aspects, suggesting that these occupations involve differing levels of occupational stress, regardless of whether this is physical, ergonomic, biological, or chemical in nature. Therefore, QOL and WA can be differentiated for taxi drivers and motorcycle taxi drivers, indicating different levels of health, and providing important data for professional groups, unions, and government institutions [3,4,5,6,7,8,9,10,11,12].

In this way, this study aimed to compare the QOL and WA (evaluated by the Work Capacity Index) between taxi and motorcycle taxi drivers, in addition to examining the correlations between QOL and WA.

## 2. Materials and Methods

A cross-sectional descriptive study was conducted in a city in the state of Goiás, Brazil. The research was accepted by the Research Ethics Committee, with respect to research ethics in studies involving humans, and approved by Ruling No. 1707302. All participants signed an informed consent form.

The population comprised 584 motorcycle taxi drivers and 70 registered taxi drivers working in the city where the study was conducted. OpenEpi® (Open Source Epidemiologic Statistics for Public Health, Atlanta, GA, USA) obtained a minimum sample of 232 motorcycle taxi drivers and 59 taxi drivers at a 95% confidence level (equation: *n* = [EDFF × N × p(1 − *p*)]/[(d^2^/Z^2^_1 − α/2_(N − 1) + *p* × (1 − *p*)]; EDFF: Drawing effect, N: population size, *p*: frequency hypothesis of the result factor in the population, d: confidence level, Z: 1.96, α: 5). The exclusion criteria were having a physical disability, being exclusively engaged in administrative activities, being on leave, having less than six months experience in the profession, and not having completed the questionnaires. Up to three attempts were made to collect data at all taxi and motorcycle taxi driver points. The final sample comprised 232 motorcycle taxi drivers and 60 taxi drivers, who were all male and aged between 20 and 60 years. The data were collected by six evaluators duly trained for this purpose. 

Data collection occurred simultaneously in both professional categories. The workers were evaluated during their breaks with due consideration of confidentiality and privacy. The questionnaires were distributed in person by previously trained researchers in the participants’ respective workplaces (taxi/motorcycle rank or taxi stand) in the intervals between runs without impeding drivers’ work. To this end, three enveloped instruments were used: a sociodemographic and occupational questionnaire; a specific instrument to evaluate WA, the Work Capacity Index (WCI); and the generic QOL instrument, the World Health Organization (WHO) Quality of Life–Bref (WHOQOL-Bref). To minimize social desirability bias, the formal consent document guaranteed anonymity, and assurance regarding anonymity was provided. The self-completion mode was used to minimize this bias. 

The first instrument was a structured questionnaire with categorized nominal data built by the authors to collect sociodemographic information. After its elaboration, the questionnaire was refined by three judges who were either researchers in the thematic area of this study or experts in the proposed method. After refinement and corrections, two pre-tests were conducted to make adjustments that facilitated the preparation of the final version used in this study.

The second instrument was the WCI, which evaluated individual WA. It was proposed by Tuomi et al. [13] and translated and validated by Martinez, Latorre, and Fischer [14]. The WCI involves relationships between physical and mental professional demands and the worker’s health status in association with their capacity to perform their work activities in the present and in the future.

The questionnaire contained 10 questions synthesized into seven dimensions: 1—current work capacity compared with the best in all their life, represented by a score of 0 to 10 points; 2—work capacity in relation to work demands, represented by two questions on the nature of work (physical, mental, or mixed), which, after weighting, provided a score of 2 to 10 points; 3—current number of diseases, both self-reported and diagnosed by a physician, obtained from a list of 51 diseases, yielding a score of between 1 and 7 points; 4—estimated loss of working hours due to diseases, obtained from a question with a score ranging from 1 to 6 points; 5—sickness absenteeism, obtained from a question on the number of absences and categorized into five groups with a score ranging from 1 to 5 points; 6—self-prognosis about work capacity, obtained from a question with a score of 1, 4, or 7 points; and 7—mental resources with a score of 1 to 4 points, obtained after weighting the answers to three questions. The assessment of the WCI, through self-perceived health, offers information about each worker’s ability, respecting individual differences and offering support measures. The results yield scores of between 7 and 49 points with scores from 7 to 27 indicating low work capacity, scores from 28 to 36 indicating moderate work capacity, scores from 37 to 43 indicating good work capacity, and scores from 44 to 49 indicating excellent work capacity [8,9]. Instructions for calculating the scores can be found in Tuomi et al. [13].

The third instrument was the WHOQOL-Bref, which was created by the WHO in 1998 and validated in Portuguese by Fleck et al. [12]. This questionnaire contains 26 Likert-type questions divided into four domains: Domain 1: physical, Domain 2: psychological, Domain 3: social relations, and Domain 4: environmental [12]. The final scores range between 20 and 100 points. The higher the score achieved, the better the QOL. The guidelines and syntax provided by the WHO were followed for the calculation of the score. The WHO did not create any categorizations for this instrument.

For statistical purposes, only the completed WHOQOL-Bref and WCI questionnaires were considered. For the sociodemographic questionnaire, all complete questionnaires were considered, and the others were discarded. All three questionnaires were discarded in cases where the respondent had one questionnaire that could not be considered.

The data obtained were doubly entered in an Excel spreadsheet and analyzed with the Statistical Package for Social Sciences (SPSS) version 22.0 (Statistical Package for the Social Sciences, IBM company, Armonk, NY, USA) and STATA 14.0 (StataCorp LLC, College Station, TX USA) for the initial descriptive analysis. Continuous variables are presented as means and standard deviations (SD), and qualitative variables are presented as absolute and relative frequencies.

The Kolmogorov-Smirnov test with Lilliefors correction was used to test the normality of the continuous variables.

The Mann-Whitney test was used to compare the categories of the independent variables in relation to the domains of QOL, the general QOL score, and the WCI. For the bivariate analysis of continuous data (domains of QOL, overall QOL score, and WCI), Spearman’s correlation coefficient was performed.

In the bivariate analysis of the nominal data, Fisher’s Exact and Pearson’s chi-square tests were used to compare the frequency of data between the two professional populations, having estimated the prevalence and gross prevalence ratio. The variables that presented a *p*-value < 0.20 in the bivariate analysis were entered into the multiple regression bivariate analysis, which was performed using the robust Poisson regression model. To verify possible factors of association/prediction for the domains of QOL and WCI, a binary logistic regression was performed in which the model was based on occupation (taxi and motorcycle taxi drivers), and, to verify the fit of the models, the Hosmer and Lemeshow statistic was used in the logistic model. 

In all tests, *p*-values < 0.05 were considered statistically significant.

## 3. Results

Cronbach’s α was calculated to verify the reliability of the two instruments in the study population, determining α = 0.82 for the WCI and α = 0.86 for the WHOQOL-Bref.

Eighty motorcycle taxi drivers and 13 taxi drivers were injured during work. Although there was no association of occupation with the occurrence of accidents (*p* = 0.058), the risk of accidents for motorcycle taxi drivers was 1.9 times higher. 

As for a place to rest during a workday, 8 motorcycle taxi drivers and 9 taxi drivers reported having a specific place, whereas 224 motorcycle taxi drivers and 51 taxi drivers did not have one. According to Fischer’s exact test, the number of taxi drivers with a place to rest was statistically higher (*p* = 0.001). 

Table 1 shows the data and comparisons for age, length of a workday in hours, number of workdays per week, and number of work accidents. According to this table, motorcycle taxi drivers were younger (*p* = 0.000), worked fewer hours per day (*p* = 0.000), and experienced more work accidents (*p* = 0.026) than taxi drivers.

Table 2 shows mean values and comparisons between QOL domains and the WCI dimensions. Motorcycle taxi drivers presented worse evaluation scores in the physical and psychological domains and QOL general scores, and better evaluation scores in the environmental QOL domains.

Table 3 compares the frequencies of the WCI classification between motorcycle taxi drivers and taxi drivers. As shown in this table, taxi drivers had substantially higher evaluation scores, such as for excellent WCI, when compared with motorcycle taxi drivers. Logistic regression revealed that being a taxi driver was a predictor of a good WCI (β = 1.901, 95% CI: 0.068–0.331 and *p* = 0.000).

Table 4 shows the logistic regression analysis used to verify QOL prediction according to occupation. These results indicate that being a taxi driver is a predictor of better physical and environmental domain and QOL general scores. The same analysis was used to verify WCI prediction according to occupation (Table 5), showing that being a taxi driver was a predictor of better results in dimensions 2, 3, and 4.

Table 6 shows that there are positive correlations between the physical domain and dimensions 1, 2, 3 and the WCI general score; the psychological domain and dimensions 2, 3, 4, 6, 7 and the WCI general score; the social domain and the WCI general score; and the QOL general score and dimension 2 and the WCI general score. There was also a negative correlation between the environmental domain and dimensions 2 and 3 and the WCI general score.

## 4. Discussion

This study evaluated the QOL and work capacity of taxi and motorcycle taxi drivers, who are workers present in most cities in Brazil and globally, using the WHOQOL-Bref and WCI instruments, respectively.

There was no difference in the frequency of accidents between the two categories of workers. However, the risk of accidents was higher among motorcycle taxi drivers. Working as a taxi driver on a motorcycle is undoubtedly more dangerous than when driving a four-wheeled vehicle because of balance issues and the lack of protective safety gear. Although accident risk was not analyzed, the specialized literature is broad and robust with an emphasis on the severity of trauma; for the motorcyclist, there are no protections similar to those for occupants of four-wheeled vehicles. In a collision, which is one of the most common types of motorcycle accident, the motorcyclist’s body absorbs all the energy generated by the impact, whether in contact with the road or with other motorcycles or motor vehicles, with consequent polytrauma [13,14,15,16,17].

The number of motorcycle taxi drivers has increased in all regions of Brazil because of either the lower cost of a motorcycle compared with a car, or the greater demand for their services, which are also less costly. However, these workers have less organizational and structural support in relation to taxi drivers and do not have a place to rest between journeys, usually staying on the sidewalk or in a garage without ventilation or comfort. Taxi drivers can rest in their car and, as a professional group that emerged earlier, are more organized and have better places to wait for their clients [18,19]. In addition, taxi drivers are more experienced, work fewer hours per week, and have a lower number of work accidents. Motorcycle taxi drivers are younger and, therefore, have less experience, maturity, and fear of accidents, making them less afraid of dangerous situations when riding a motorcycle. Additionally, a motorcycle does not provide structural protection for motorcyclists [20].

Taxi and motorcycle taxi drivers are professionals predisposed to health risks with a consequent impact on QOL, especially in the physical domain. In this sense, the present study showed that motorcycle taxi drivers had worse evaluation scores in the physical domain compared to taxi drivers. This fact can be related to the negative impact of the profession on the seven facets evaluated in this domain: pain and discomfort, energy and fatigue, sleep and rest, mobility, activities of daily living, dependence on medication or treatments, and ability to work.

Motorcycle taxi drivers do not always have physical conditioning of the specific muscles involved in a riding posture which ends up generating pain, discomfort, and fatigue in the lumbar and cervical/head regions in addition to pain in the upper and lower limbs. These are allied to stress and inherently function to negatively impact rest/sleep, making self-medication common among this group [7,8,9,10,11,19]. 

This is associated with the fact that the average age of motorcycle taxi drivers is lower than that of taxi drivers, which produces the phenomenon of lack of labor limits; thus, they work more days per week and, consequently, are predisposed to greater occurrence of accidents. Such findings have a negative impact on welfare and QOL and a negative overall impact on workers’ life and health. Taxi drivers usually work long hours, which can result in less time for resting or termination of employment due to the working period, thus presenting a worse prognosis in the environmental domain, as verified by the WCI questionnaire [7,14,18].

These findings are confirmed in a study of motorcycle taxi drivers in another city in the central-west region of Brazil in which many professionals reported discomfort during work. Drivers with a certain type of diagnosed disease had a lower self-perceived QOL in relation to physical functioning, body pain, and general health than drivers without a diagnosed disease [19,20].

Motorcycle taxi drivers also presented with worse evaluation scores on the psychological (positive feelings, thinking, learning, memory and concentration, self-esteem, body image and appearance, negative feelings, spirituality/religion/personal beliefs) and environmental (physical security and protection, home environment, financial resources, care health and social: availability and quality, opportunities to acquire new information and skills, participation in recreation/leisure, physical environment, transportation) domains. The precarious working conditions, long journeys and work overload, greater length of exposure to serious risks (accidents) in the activity, professional devaluation, lack of occupational perspective, monotony, stress, anxiety, concern, constant/uninterrupted need for level of self-attention/self-control/concentration, pressure for time, urban violence, irritability, and productivity can generate work stress and neurophysiological disorders, affecting mental efficiency and motivation for work. All these factors can negatively impact the evaluation scores in the psychological and environmental domains as well as the overall QOL score [7,8,9,10,11,21,22].

As for the WCI, motorcycle taxi drivers had a lower evaluation scores in dimensions 1 to 4 and in the WCI general score. They had lower perceptions of work capacity (dimension 1), more physical and mental demands (dimension 2), more diagnosed diseases (dimension 3), greater incapacity to perform work (dimension 4), and a worse WCI evaluation related to the total scores in the seven dimensions. Another study showed that the individual’s perception of WA is associated with work-related factors and can also be related to the perception of QOL inside and outside the work environment [23].

Working conditions for motorcycle taxi drivers are naturally unsafe as they are more exposed to changes in the weather, solar radiation, noise, and traffic dangers; have higher psychological demands needing more concentration; have lower remuneration and no employment bonds or guarantees; and work in a fast-paced environment with inadequate ergonomic conditions [22,23,24,25,26].

A study by Meng et al. [27] showed that drivers have more frequent episodes of fatigue mostly due to long driving hours. Taxi drivers had a better WCI evaluation score than motorcycle taxi drivers.

Additionally, the results showed that working as a taxi driver is a predictor of a good WCI evaluation score and better physical and environmental domain and QOL general scores when compared with working as a motorcycle taxi driver; however, the results are specific to an inner city. This finding is consistent with previous findings and reaffirms that motorcycle taxi drivers have deficits in physical safety and protection, home environment, financial resources, health and social care, availability and quality, opportunities to learn new information and skills, participation in/opportunities for leisure activities, physical environment (pollution/noise/traffic/climate), and transport. All these items are related to the environmental domain. They also experience greater pain/discomfort, less energy/fatigue, worse sleeping/resting periods, less mobility, difficulties in daily life activities, addiction to medications or treatments, and worse work capacity. All these items are related to the physical domain [12,26,28].

As for the WCI, the results showed that working as a taxi driver is a predictor of better WCI general scores, better physical and mental demands (dimension 2), fewer diagnosed diseases (dimension 3), and less incapacity to perform work (dimension 4).

The findings in the present study justify and support those of other studies that have reported increased cardiovascular and genitourinary risks for motorcycle taxi drivers. Although this study did not specifically evaluate such variables, the results showed a worse evaluation of aspects related to health and work capacity, which directly impact the health–work relationship. Gas inhalation, poor working conditions, noise, and poor sleep quality result in cardiovascular risks and increased incidence of systemic arterial hypertension and arterial occlusion. Sitting on a warm seat for long hours can result in genitourinary conditions, such as infections and trauma [28,29,30,31,32,33].

The correlations between QOL and the WCI scores confirm the findings and the perspectives of each domain and dimension as recommended by certain studies that analyzed them separately, confirming the relevance of using these two instruments simultaneously to evaluate different work categories and occupations [34,35,36]. A healthy and harmonious work environment is important to maintain and develop high-quality work and improve employees’ lives. It is important to reduce work fatigue, stress, and the imbalance between work and family [34]. Workers who are pleased with their jobs work with more attention, precision, and productivity; have reduced occupational and work-related risks; protect themselves against risky situations; and help protect their colleagues by creating an unconscious care network among workers, leading to higher productivity, lower rates of disease/work accident leave, and, consequently, lower costs for employers and the public health system [32,33,34,35,36].

In this context, there are significant differences related to QOL and the health of individual urban transport workers, revealing the importance of studying and analyzing this occupational group more accurately to propose policies and improvements, thereby promoting health and better work performance.

The implications of this study are important for the professional categories considered (especially the category of motorcycle riders, who had the worst evaluation scores), who will be able to review the organizational/labor structure they offer to their workers, such as workload, training/improvement opportunities, leisure/rest opportunities, and review of the employment modalities. The findings will also serve as support for these professional categories in seeking from the competency bodies, prevention policies and better health care (promotion/education and prevention campaigns) laws that ensure social security and new labor standards for the profession, consistent with contemporary labor demands. In addition, this research suggests that other aspects must be investigated in these professional categories since the evaluations of QOL and WA suggest alterations in general health that can lead to serious health, family, social, and behavioral consequences. Because it is a profession that deals with other people, the negative impacts on these workers certainly have consequences for the public, with serious implications for the health of taxi-users, since accidents involving motorcycles usually cause moderate to severe injuries.

The occupational factors to which motorcycle taxi drivers are exposed, concerning physical, ergonomic, chemical, and biological risks, do not differ depending on the locality (country). Those aspects that can change are the legislation and organization of the work; therefore, the results obtained in this study can be considered by countries that have this professional activity aimed at further investigation, including health/QOL analysis of these workers, and health care and work in this professional category [3,4,5,6,7,8,15,17,21].

The advantages of the methods utilized in this study are related to the worldwide comparability of the questionnaire results, since the tools used are international and they measure variables that are important from a health perspective, but also from social, labor, environmental, and mental perspectives. In general, questionnaires are objective; they are easier and quicker for people to answer, the answers of different respondents are easier to compare, peoples’ answers are easier to analyze, response choices can clarify the question’s meaning for respondents, people are more likely to provide answers on sensitive topics, they inspire fewer irrelevant or clouded answers, less articulate or less literate respondents are not at a disadvantage, replication is easier, and they allow the interpreter to assess guests’ prior knowledge base and feelings. The negative points are that questionnaires, like many evaluation methods, occur after the event, so participants may forget important issues. Questionnaires are standardized, so it is not possible to explain any points in the questions that participants might misinterpret. Volunteers may not be willing to answer the questions, might not wish to reveal the information, or might think that they will not benefit from responding, or perhaps even be penalized for giving their real opinion. Respondents should be told why the information is being collected and how the results will be beneficial, asked to reply honestly, and told that, if their response is negative, it is just as useful as a more positive opinion [37,38,39,40]. 

To minimize social desirability bias, important in research with questionnaires, anonymity was guaranteed in the formal consent document, and assurance was provided regarding anonymity. In addition to using and ensuring anonymity, the self-completion mode was also used. It would be interesting to repeat the data collection a few days after the first collection, but this practice is difficult and expensive, and ensuring the volunteers’ acceptance is difficult, which would probably reduce the number of responses. Since the motorcycle taxi drivers demonstrated worse evaluation scores on the variables, it is believed that there was no social desirability bias for this population, and the literature corroborates the findings [8,9,11,15,17,18,21,22,25,26,27,28,29,30,31,32,33]. To eliminate this bias, the questionnaire could be delivered together with the others to provide verification [41].

A limitation of this study was the use of instruments not specific to the study population. However, there are no specific instruments to evaluate the variables of the questionnaires used in the study population; the questionnaires do not make it possible to specify the points of greatest satisfaction or dissatisfaction regarding their health, social, labor, and environmental contact, so the analysis and the interventions to be taken cannot be individualized. Further studies using a larger sample and other countries are needed to point to the general profile of the QOL and work capacity of the two professional classes and to verify that, in all countries, the motorcycle class has a worse evaluation of these variables, making it possible for the WHO and International Labour Organization to suggest global changes aimed at improving their health and work.

## 5. Conclusions

Motorcycle taxi drivers had worse evaluation scores on the physical (pain and discomfort, energy and fatigue, sleep and rest, mobility, activities of daily living, dependence on medication or treatments, and work capacity), psychological (positive feelings, self-esteem, body image and appearance, negative feelings, and spirituality/religion/personal beliefs), and environmental domains (physical security and protection, home environment, financial resources, health and social care—availability and quality, opportunities for acquiring new information and skills, participation in recreation/leisure, physical environment, and transportation) and general evaluation of the QOL. Regarding the WA, motorcycle taxi drivers had lower self-perceived work capacity (dimension 1), greater physical and mental demands (dimension 2), more pathological conditions that had already been diagnosed (dimension 3), higher inability to pursue the profession (dimension 4), and worse overall WA (WCI). It is also concluded that the QOL and the WA are related, so the change of some aspect linked to either one of these concepts is likely to impact the other.

## Figures and Tables

**Table 1:** Comparisons of age, length of workday in hours, number of workdays per week, and number of work accidents between taxi and motorcycle taxi drivers.

Variables	Occupation	N	Mean ± SD	*p*-Value
Age	Motorcycle taxi drivers	232	31.26 ± 15.86	0.000
	Taxi driver	60	39.98 ± 10.59	
Length/hours	Motorcycle taxi drivers	232	11.45 ± 2.46	0.000
	Taxi driver	60	24.00 ± 0.00	
Number of workdays	Motorcycle taxi drivers	232	6.28 ± 0.72	0.142
	Taxi driver	60	6.01 ± 1.38	
Number of work accidents	Motorcycle taxi drivers	186	0.70 ± 1.12	0.026
	Taxi driver	60	0.38 ± 0.90	

SD: standard deviation.

**Table 2:** Mean values and comparisons between quality of life (QOL) domains and Work Capacity Index (WCI) dimensions.

Variables	Occupation	Mean ± SD	*p*-Value
Physical domain	Motorcycle taxi drivers	77.89 ± 11.27	0.000
	Taxi driver	87.02 ± 6.60	
Psychological domain	Motorcycle taxi drivers	72.73 ± 10.03	0.001
	Taxi driver	76.80 ± 7.292	
Social domain	Motorcycle taxi drivers	76.68 ± 11.93	0.118
	Taxi driver	79.02 ± 9.76	
Environmental domain	Motorcycle taxi drivers	61.69 ± 10.78	0.000
	Taxi driver	54.68 ± 5.32	
QOL general score	Motorcycle taxi drivers	72.25 ± 6.61	0.018
	Taxi driver	74.38 ± 4.29	
Dimension 1 (Current capacity for work)	Motorcycle taxi drivers	9.88 ± 0.33	0.008
	Taxi driver	10.00 ± 0.03	
Dimension 2 (Physical and mental demands)	Motorcycle taxi drivers	8.58 ± 0.54	0.000
	Taxi driver	9.23 ± 0.76	
Dimension 3 (Diagnosed diseases)	Motorcycle taxi drivers	1.40 ± 1.21	0.000
	Taxi driver	2.15 ± 1.49	
Dimension 4 (Incapacity to work)	Motorcycle taxi drivers	5.81 ± 0.43	0.054
	Taxi driver	5.93 ± 0.25	
Dimension 5 (Absenteeism)	Motorcycle taxi drivers	4.98 ± 0.13	0.307
	Taxi driver	5.00 ± 0.02	
Dimension 6 (Self-prognosis)	Motorcycle taxi drivers	6.80 ± 0.72	0.954
	Taxi driver	6.80 ± 0.75	
Dimension 7 (Mental resources)	Motorcycle taxi drivers	3.88 ± 0.32	0.090
	Taxi driver	3.80 ± 0.40	
WCI general score	Motorcycle taxi drivers	41.36 ± 1.67	0.000
	Taxi driver	42.91 ± 2.12	

Comparisons undertaken with the Mann-Whitney test. QOL: quality of life. WCI: Work Capacity Index.

**Table 3:** Comparisons of the WCI classifications between motorcycle taxi drivers and taxi drivers.

WCI Classification	Motorcycle Taxi Drivers	Taxi Driver	95% CI	*p*-Value
Moderate	4 (1.7%)	1 (1.7%)	0.640–1.761	0.969 *
Good	215 (92.7%)	42 (70.0%)	1.121–1.213	0.001 **
Excellent	13 (5.6%)	17 (28.3%)	1.384–1.752	0.001 ***

Comparisons undertaken with Fisher’s Exact test or Pearson’s chi-square test. CI: confidence interval. WCI: Work Capacity Index. * Prevalence ratio (RP) = 1.03; ** RP = 1.39; *** RP = 1.2.

**Table 4:** Binary logistic regression analysis for the QOL domains and overall QOL score according to occupation.

Groups of Variables	β	*p*-Value	OR	β 95% CI	
**Physical domain**					
Taxi drivers	0.089	0.000	1.093	1.047	1.142
Motorcycle taxi drivers (ref.)		-	1		
**Psychological domain**					
Taxi drivers	0.008	0.678	1.008	0.969	1.049
Motorcycle taxi drivers (ref.)		-	1		
**Social domain**					
Taxi drivers	−0.010	0.550	0.991	0.960	1.022
Motorcycle taxi drivers (ref.)		-	1		
**Environmental domain**					
Taxi drivers	0.055	0.002	0.947	0.914	0.980
Motorcycle taxi drivers (ref.)		-	1		
**QOL general score**					
Taxi drivers	0.058	0.020	1.059	1.009	1.112
Motorcycle taxi drivers (ref.)		-	1		

Binary logistic regression analysis. QOL: quality of life. Ref: Reference group in the regression model. OR: odds ratio. CI: coefficient interval. Hosmer and Lemeshow test = 0.216.

**Table 5:** Binary logistic regression analysis for the WCI domains according to occupation.

Groups of Variables	β	*p*-Value	OR	β 95% CI	
**Dimension 1** (Current capacity for work)					
Taxi drivers	20.053	0.998	51.584	0.321	3.437
Motorcycle taxi drivers (ref.)		-	1		
**Dimension 2** (Physical and mental demands)					
Taxi drivers	1.592	0.000	4.916	2.775	8.708
Motorcycle taxi drivers (ref.)		-	1		
**Dimension 3** (Diagnosed diseases)					
Taxi drivers	0.512	0.000	1.669	1.261	2.209
Motorcycle taxi drivers (ref.)		-	1		
**Dimension 4** (Incapacity to work)					
Taxi drivers	2.430	0.022	11.362	1.413	91.370
Motorcycle taxi drivers (ref.)		-	1		
**Dimension 5** (Absenteeism)	19.076	0.999	19.316	0.245	0.765
Taxi drivers		-	1		
Motorcycle taxi drivers (ref.)					
**Dimension 6** (Self-prognosis)					
Taxi drivers	−0.645	0.077	0.525	0.257	1.072
Motorcycle taxi drivers (ref.)		-	1		
**Dimension 7** (Mental resources)					
Taxi drivers	−0.969	0.088	0.379	0.125	1.153
Motorcycle taxi drivers (ref.)		-	1		
**WCI general score**					
Taxi drivers	0.535	0.000	1.708	1.403	2.080
Motorcycle taxi drivers (ref.)		-	1		

Binary logistic regression analysis. Ref: Reference group in the regression model. WCI: Work Capacity Index. OR: odds ratio. CI: coefficient interval. Hosmer and Lemeshow test = 0.591.

**Table 6:** Correlations between QOL and the WCI using Spearman’s correlation coefficient.

QOL	Statistics	Dimension 1	Dimension 2	Dimension 3	Dimension 4	Dimension 5	Dimension 6	Dimension 7	WCI General
Physical domain	Correlation	0.151 *	0.250 *	0.226 *	0.031	0.058	0.113	−0.029	0.293 *
	*p*-value	0.010	0.000	0.000	0.596	0.323	0.053	0.628	0.000
Psychological domain	Correlation	0.096	0.160 *	0.177 *	0.118 *	0.005	0.156 *	0.134 *	0.277 *
	*p*-value	0.103	0.006	0.002	0.044	0.938	0.007	0.022	0.000
Social domain	Correlation	0.037	0.039	0.062	0.040	−0.020	0.080	0.006	0.117 *
	*p*-value	0.529	0.502	0.290	0.493	0.740	0.172	0.914	0.046
Environmental domain	Correlation	−0.103	−0.176 *	−0.294 *	−0.033	−0.086	−0.085	−0.005	−0.310 *
	*p*-value	0.078	0.003	0.000	0.580	0.143	0.149	0.938	0.000
QOL general score	Correlation	0.100	0.165 *	0.088	0.073	−0.030	0.103	0.045	0.185 *
	*p*-value	0.088	0.005	0.133	0.212	0.611	0.080	0.441	0.001

WCI: Work Capacity Index. QOL: quality of life. * *p* < 0.05 according to the Spearman’s correlation test. Dimension 1: Current capacity for work; Dimension 2: Physical and mental demands; Dimension 3: Diagnosed diseases; Dimension 4: Incapacity to work; Dimension 5: Absenteeism; Dimension 6: Self-prognosis; Dimension 7: Mental resources.
